# Cardiac mri is useful in icd patients undergoing ablation for ventricular tachycardia

**DOI:** 10.1186/1532-429X-13-S1-P253

**Published:** 2011-02-02

**Authors:** Melissa Robinson, Francis Marchlinski, Harold Litt

**Affiliations:** 1 University of Illinois at Chicago, Chicago, IL, USA; 2 University of Pennsylvania Medical Center, Philadelphia, PA, USA

## Introduction

Catheter ablation is used to control ventricular arrhythmias (VT/VF) in a multitude of substrates. MR can delineate regions of scar and may guide ablation procedures, however the presence of an ICD has been an issue both for safety and MR image quality. Recent studies have demonstrated acceptable safety for scanning of device patients in certain circumstances, but efficacy has not been evaluated.

## Purpose

To evaluate the clinical utility of CMR in patients with ICDs planned for VT ablation.

## Methods

Consecutive VT ablation patients with ICDs referred for CMR between 07/08 and 05/10 were evaluated. Informed consent was obtained and patients imaged at 1.5T. The ICD was interrogated before and after the study, with tachycardia detection and therapy disabled and back-up pacing programmed during scanning.

## Results

Forty-two patients with ICDs underwent CMR from July 2008 and May 2010 (24% women; mean 54 ± 14 yr; mean LV EF 41 ± 20%). The most common diagnosis was dilated cardiomyopathy (36%), with ARVD/C and ischemic cardiomyopathy in 19% each. Defibrillators were 35 ± 28 months old, leads 44 ± 38 months. No complications of scanning were seen and post-procedure device parameters (thresholds, impedance and battery voltage) were unchanged. Patients underwent CMR a mean of 3.7 ± 5 days pre-ablation (28/42). Six patients were scanned in anticipation of ablation, but never received one; three because of normal CMRs, the other three because of clinical condition. Eight patients (3 DCM, 4 ICM and 1 HCM) were done after non-inducibility at EP study or normal endocardial voltage maps. Basal septal midmyocardial delayed enhancement (DE) was seen in 6/8 of these.

DE correlated well qualitatively with electroanatomic mapping in 30/31 pts, with one pt having normal endocardial and epicardial maps, but transmural inferolateral DE. Two patients underwent upfront epicardial ablations based on CMR findings and two normal studies facilitated the decision for device extraction after ablation. Three patients with HCM and midmyocardial scar were referred for surgical ablation after unsuccessful endo- and epicardial ablations. There was a trend toward lower acute success with ablation when mid-myocardial scar was seen (61% with v. 89% without; p=0.10).

## Conclusions

In this series of 42 patients with ICDs planned for VT ablation, cardiac MR was performed safely and results significantly impacted patient care. Mid-myocardial scar may be a marker for lower success with current ablation strategies and is a frequent finding in patients with non-inducibility or normal endocardial voltage maps.

